# Tunnelled Central Venous Catheter-Related Problems in the Early Phase of Haematopoietic Stem Cell Transplantation and Effects on Transplant Outcome

**DOI:** 10.4274/tjh.2013.0278

**Published:** 2015-02-15

**Authors:** Mahmut Yeral, Can Boğa, Levent Oğuzkurt, Hikmet Eda Alışkan, Hakan Özdoğu, Yusuf Ziya Demiroğlu

**Affiliations:** 1 Başkent University Faculty of Medicine, Adana Adult Bone Marrow Transplantation Centre, Department of Hematology, Adana, Turkey; 2 Başkent University Faculty of Medicine, Department of Radiology, Ankara, Turkey; 3 Başkent University Faculty of Medicine, Department of Medical Microbiology, Ankara, Turkey; 4 Başkent University Faculty of Medicine, Department of Infectious Disease and Clinical Microbiology, Ankara, Turkey

**Keywords:** Tunnelled central venous catheter, Haematopoietic stem cell transplantation, Thrombosis, Infection

## Abstract

**Objective:**

Haematopoietic stem cell recipients need central venous catheters (CVCs) for easy administration of intravenous fluid, medications, apheresis, or dialysis procedures. However, CVCs may lead to infectious or non-infectious complications such as thrombosis. The effect of these complications on transplantation outcome is not clear. This manuscript presents the complication rates of double-lumen tunnelled CVCs and their effect on transplantation outcome.

**Materials and Methods:**

Data from 111 consecutive patients, of whom 75 received autologous and 36 received allogeneic peripheral blood stem cell transplantations, were collected retrospectively. The data were validated by the Record Inspection Group of the related JACIE-accredited transplantation centre.

**Results:**

Thrombosis developed in 2.7% of recipients (0.9 per 1000 catheter days). Catheter-related infection was identified in 14 (12.6%) patients (3.6 per 1000 catheter days). Coagulase-negative Staphylococcus was the most common causative agent. Engraftment time, rate of 100-day mortality, and development of grade II-IV graft-versus-host disease were not found to be associated with catheter-related complications.

**Conclusion:**

These results indicate that adverse events related with tunnelled CVCs are manageable and have no negative effects on transplant outcome.

## Abstract (tr)

**Amaç:**

Hematopoetik kök hücre nakli alıcıları damar içi mayi, ilaç, aferez ve diyaliz işlemleri için santral venöz kateterlere (SVK) ihtiyaç duyarlar. Ancak SVK’ların enfeksiyöz ve tromboz gibi enfeksiyöz olmayan komplikasyonları vardır. Bu komplikasyonların nakil sürecine etkisi net değildir. Bu çalışma ile çift lümen tünelli santral kateter komplikasyonlarının sıklığı ve nakil sürecine etkisi araştırılmıştır.

**Gereç ve Yöntemler:**

Geriye dönük olarak 75 otolog ve 39 allojeneik periferik kök hücre nakli uygulanan toplam 111 hastanın verileri toparlandı. Verilerin doğruluğu JACIE akredite merkezimizin kayıt denetleme grubu tarafından kontrol edildi.

**Bulgular:**

Olguların %2,7’sinde tromboz gelişti (1000 kateter gününde 0,9). Hastaların 14’ünde (%12,6) kateter ilişkili enfeksiyon tesbit edildi (1000 kateter gününde 3,6). Koagulaz negatif Stafilokoklar en sık etken idi. Kateter ilişkili komplikasyonlar ile engrafman zamanı, evre II-IV graft versus host hastalığı ve 100 günlük mortalite arasında bir ilişki saptanmadı.

**Sonuç:**

Sonuçlarımız nakil hastalarında tünelli SVK komplikasyonlarının yönetilebilir olduğunu ve bu komplikasyonların nakil üzerine olumsuz bir etki yaratmadığını göstermektedir.

## INTRODUCTION

Haematopoietic stem cell recipients need central venous catheters (CVCs) for chemotherapy, parenteral nutrition, and blood infusion. A wide range of catheter types can be used. However, the type, number of lumens, diameter, and insertion site of the catheter should be appropriate for the procedure, as these affect the frequency of CVC-related complications [1,2,3]. Tunnelled CVC is considered appropriate for neutropenic and bone marrow transplant patients. Double-lumen tunnelled CVCs have a lower risk of infection than non-tunnelled catheters [4]. These are long-term catheters that allow for the simultaneous infusion of physically incompatible drugs, blood products, and stem cells. Long-term, continuous total parenteral nutrition can be provided via these catheters. There is limited literature on the complications of tunnelled CVC in haematopoietic stem cell recipients. The effects of these complications on the outcome parameters of transplant recipients remain to be identified.

This study aimed to determine the frequency of tunnelled CVC complications in patients who underwent peripheral blood stem cell transplantation and to analyse the effects of complications on engraftment time, 100-day mortality, and acute graft-versus-host disease (GVHD).

## MATERIALS AND METHODS

### Study Plan

This was a retrospective, cross-sectional study; it included 111 patients who underwent haematopoietic peripheral blood stem cell transplantation between May 2011 and January 2013.

Data were collected from a previously authorised and validated Hospital Information Management System (Nucleus, Monad, Ankara, Turkey). The data were validated by the Record Inspection Group of the related department. 

The primary endpoint of the study was the rate of catheter-related complications. Haemorrhage, thrombosis, and infection data obtained from the record system were calculated according to the number of catheterisations and per 1000-catheter days. Secondary endpoints were the effects of complications on thrombocyte and neutrophil engraftment, 100-day mortality, and development of acute GVHD.

The study included patients who were considered eligible for autologous and allogeneic peripheral blood haematopoietic stem cell transplantation by the clinical directors of the transplant centre. Patient eligibility was evaluated according to the Republic of Turkey Regulations on Tissue and Stem Cell Transplantation and FACT-JACIE Standards (version 5.2) [5].

Patients who had a purulent skin infection of the chest or arm during catheter application, those who had chest trauma, those who received radiotherapy in the chest region, and those of paediatric age (<16 years) were excluded from the study. The study was approved by the Başkent University Research Committee.

### Timing of Placement, Replacement, or Removal of Catheters

Tunnelled CVCs were placed in patients just prior to starting a conditioning regime for peripheral blood haematopoietic stem cell transplantation. Naturally, these CVCs were not used for harvesting peripheral blood cells. Clinicians continually reviewed the need for central venous access in individual patients. Catheters were replaced only for clinical indications like clinical infection or purulence at the insertion site.

### Selection and Placement of Catheters

All CVCs were inserted in the Interventional Radiology Unit by a radiologist or a specialised nurse only. Complete blood count and coagulation tests were performed before the procedure for all patients who received a double-lumen, 18-cm-long, tunnelled CVC (Cath, Medcomp, Harleysville, PA, USA). Prophylactic antibiotics at the time of insertion of the CVC, in-line filters, or anti-infective/microbial lock prophylaxis were not used.

The Interventional Radiology Unit inserted CVCs using an ultrasound image-guided percutaneous technique. The right internal jugular vein was preferred for first access; however, the left internal jugular was used in the case of a problem on the right side. The vein was punctured with an 18-G needle under ultrasonography guidance [6].

### Care of the Catheters

A sterile gauze dressing, secured with adhesive tape, was only to be changed by a catheter nurse until sufficient healing had taken place (following adherence of the cuff; usually within 3 weeks of insertion). During this period, the gauze was changed by the catheter nurse at least every 48 h. Flushing was used after placement prior to and after fluid infusion or injection, and prior to and after blood drawing.

The patients carried responsibility for the catheter following hospital discharge.

### Culture Collection Procedure

A 10-mL blood sample was collected from the peripheral vein and from each lumen of the catheter simultaneously into an aerobic blood culture bottle. Culture bottles were incubated in a BACTEC 9240 (Becton Dickinson Microbiology Systems, Sparks, MD, USA) automatic culture device.

Direct stained preparations were made from blood culture bottles, which gave a positive result from the system. The cultures used media containing 5% sheep blood and eosin methylene blue. Cultures were evaluated after incubation at 37 °C for 24-48 h.

Conventional methods and BBL crystal identification systems (Becton Dickinson Microbiology Systems) were used to identify bacteria. Antibiotic sensitivity was determined via the Kirby-Bauer disc diffusion method according to the recommendations of the Clinical and Laboratory Standards Institute.

### Identification of Complications

If the same agent grew in the blood culture collected from the catheter lumen and the peripheral blood, or if the growth in the blood culture collected from the catheter lumen occurred 2 h prior to that in peripheral blood, it was considered a catheter-related blood-stream infection. If erythema, increased temperature, or sensitivity occurred within a 2-cm periphery of the insertion site of the catheter, it was considered a catheter-exit-site infection, irrespective of whether the blood culture contained bacterial growth [7]. Thrombosis as detected by Doppler ultrasonographic examination was evaluated to obtain more objective and reliable data and thus avoid misevaluation.

### Statistical Analysis

Statistical analysis was performed using SPSS 17.0. For each continuous variable, normality was checked using Kolmogorov-Smirnov and Shapiro-Wilk tests. Comparisons between groups were applied using a one-way Student’s t-test for normally distributed data, and the Mann-Whitney test was used for data not normally distributed. Categorical variables between groups were analysed using the chi-square test.

Statistical significance was accepted at p<0.05.

## RESULTS

Transplant patients in the study included 40 females (36%) and 71 males (64%), with a median age of 49 years (range: 16-64). Of the patients, 75 (67.6%) underwent an autologous transplant while 36 (32.4%) underwent an allogeneic transplant. Diagnoses and stem cell transplant types are presented in Table 1.

All 111 catheters placed in the patients were tunnelled central catheters. Of these, 100 (90%) were placed in the right internal jugular vein and 11 (10%) in the left internal jugular vein. No catheters were placed in the subclavian veins. Catheters placed in the internal jugular veins had a median size of 10 F (range: 8-14 F). Total catheter dwell time was 3322 catheter days. Median catheter dwell time per catheter was 26 (range: 15-106) days; the median was 14 (15-85) days for autologous transplants and 30 (15-106) days for allogeneic transplants (p<0.05). Of the 111 catheters used during bone marrow transplantation, 108 (92.7%) were removed when they were no longer needed. Two catheters were removed due to infection and thrombosis complications, and 1 catheter (0.9%) exited spontaneously.

The incidences of tunnelled CVC-related complications in transplant recipients are shown in Table 2.

Analysis according to catheterisation day showed complications in a total of 26 (23.4%) patients (7.8 complications per 1000 catheter days). Mild haemorrhage, in the form of leakage, was observed in 2 patients (1.8%).

Three of 111 patients developed symptomatic thrombosis attack (2.7%), representing a rate of 0.9 per 1000 catheter days. Symptomatic thrombosis rates of the autologous and allogeneic groups were 2 (2.7%) and 1 (2.8%), respectively. Fibrin sheaths, which cause catheter dysfunction, were identified in 7 (6.3%) patients (2.1 per 1000 catheter days). There was no significant difference between the allogeneic and autologous sub-groups in terms of the frequencies of catheter-related thrombosis and fibrin sheaths (p>0.05).

Catheter-related infections were identified in 14 (12.6%) patients. Of these infections, 2 (1.8%) were considered exit-site infections. During follow-up, catheter-related blood-stream infection was detected in 12 (10.8%) patients (3.6 per 1000 catheter days), which represented 9.3% and 13.9% of autologous and allogeneic transplant recipients, respectively. One (8.4%) infection was caused by Corynebacterium jeikeium, 2 (16.6%) were caused by Escherichia coli, and the remaining 9 catheter-related blood-stream infections (75%) were caused by coagulase-negative Staphylococcus spp.

The relationship between complications and catheter days was assessed. There was no significant relationship with thrombosis (p>0.05), but there was a statistically significant relationship between duration of catheter dwelling and infection (p<0.05).

Outcome parameters according to type of stem cell transplantation are documented in Table 3.

All patients achieved absolute neutrophil counts of ≥0.5x109/L at a median of day 12 (range: 8-17) and platelet counts of ≥20x109/L at a median of day 11 (range: 8-23). In the autologous and allogeneic sub-groups, the neutrophil engraftment times were 12 (8-17) and 12 (10-17) days, respectively; thrombocyte engraftment times were 11 (8-21) and 12 (9-23) days, respectively. We observed no significant difference between the autologous and allogeneic groups in terms of engraftment time (p>0.05). Grade II-IV acute GVHD was identified in 4 (11.1%) patients who underwent allogeneic stem cell transplants. Analyses showed that infection and thrombosis did not delay engraftment times. No evidence of acute GVHD was observed in patients who developed catheter-related blood-stream infection. The overall 100-day mortality rate was 3.6% in all stem cell recipients. None of the mortalities were related to catheter complications.

## DISCUSSION

Tunnelled CVCs differ from other catheters in the sense that one part of the catheter remains in subcutaneous tissue after the venous entrance level. This tunnel theoretically provides stability and serves as a barrier against skin-related infections. If high-flow venous access is necessary for the patient, and if this vascular access is required for at least 3 weeks, a tunnelled catheter is preferred.

Catheters can have some complications. Mechanical complications in the early period include conditions such as haematoma, haemorrhage, artery adjustments, and pneumothorax. Previous studies show that, when implanted by experienced personnel using imaging techniques, the incidence of complications is low [1,6,8]. In the present study, tunnelled central catheters were inserted by experienced staff of the Interventional Radiology Unit under ultrasonographic guidance. In our study, haemorrhage in the form of leakage was observed in 2 catheters as a mechanical complication. Leakage was controlled by local compression without further intervention.

A review of the literature showed that infection levels in patients with a haematological malignity with tunnelled or non-tunnelled CVCs varied from 4.5% to 20.8% [9,10,11]. The frequency of infection in patients with haematological malignancy was higher than in those with solitary tumours [12,13]. In acute myeloid leukemia patients, the catheter-related bacteraemia rate was 31.5%, and 69.6% had a mortal course after stem cell infusion [14]. In a large patient series that included mostly acute leukemia patients (690 Hickman catheters), catheter-related bacteraemia was found in 2.9 per 1000 catheter days [15]. The incidence of Hickman catheter-related infections in cancer patients was reported to be 5.98-7.2 per 1000 catheter days [16]. In contrast, the incidence of catheter-related infections was 2.7% in intensive care unit patients who were placed with short-term CVCs (5.9 per 1000 catheter days) [17]. Our rate of catheter-related infections was lower than those in intensive care unit patients and haematological and non-haematological malignancy patients with temporary CVCs. Blood-stream infection rates were similar to those in previous reports of complications of tunnelled CVCs. Catheter infection levels in allogeneic transplant patients were higher than those in autologous catheter patients. This is likely caused by the use of immune suppressive agents in allogeneic transplants, catheter use time, and more frequent catheter manipulations. Nevertheless, there was no significant difference between the 2 groups. Two cases of exit-site infections were controlled via local treatment and care. A wide spectrum of microorganisms can cause catheter-related infections. However, the frequency of the agents varies according to catheter placement location and type. The primary source of infection is contamination from the skin flora of both the patient and healthcare personnel. It was reported that 34.1%-50% of catheter-related infections are caused by coagulase-negative Staphylococcus, while 9.9%-20% are caused by S. aureus [17,18,19,20]. In our study, most of the catheter-related infections were caused by coagulase-negative Staphylococcus, suggesting that efficient catheter care or antimicrobial prophylaxis with levofloxacin for transplantation procedure could prevent gram-negative infections.

Fibrin sheath formation around the catheter is one of the most important problems that disrupt catheter functions. This has been reported to occur in 56% of patients with short-term catheters [21,22,23]. A fibrin sheath was identified in 7 of our patients. Selective t-PA was infused into the catheter in 5 patients; mechanical intervention via a guide wire was used in the remaining 2 patients. Catheter function was re-established in all patients following the procedure.

The type of malignancy, chemotherapy type, catheter type, placement location, catheter usage errors, and inappropriate care are risk factors for thrombosis. Most cases of catheter-related thrombosis are asymptomatic. A thrombosis incidence of 17%-18% was reported in tunnelled CVCs in solid organ tumours [13,24]. The incidence of symptomatic thrombosis is 1.2%-13% in patients with a haematological malignancy and central catheter [25]. We report here much lower thrombosis rates than did previous studies on tunnelled or non-tunnelled CVCs. All 3 of our thrombosis cases developed 21 days after placement of catheters. The type of transplantation had no significant effect on the rate of complications. However, it should be noted that our series included relatively few thrombosis cases. The risk of thrombosis and infection in Hickman catheters in patients who underwent chemotherapy infusion was approximately 5 times greater than that for implanted ports [26]. Despite the lower complication rates, that type of catheter cannot be considered in haematopoietic peripheral blood stem cell transplantation, especially in allogeneic transplants. Therefore, we did not use implanted venous catheters in our patients.

Catheter-related complications, mostly infections, can lead to local or systemic inflammatory conditions. All inflammatory states may contribute to the development of GVHD. In this study, catheter-related exit-site or blood-stream infections were not remarkable observations in the allogeneic transplant recipients who developed grade II-IV GVHD. At the 100 days of follow-up, 2 patients had been lost due to disease progression, 1 due to hepatic veno-occlusive disease, and 1 due to lung infection. Catheter-related complications had no effect on the mortality rate in transplant patients.

Three catheters were removed or exited spontaneously before completion of the survey. One catheter in a patient who developed thrombosis was removed because it was not re-canalised despite thrombolytic treatment. One of the catheters exited spontaneously. The other catheter was colonised by Corynebacterium jeikeium, which is a hospital-acquired microorganism resistant to multiple antimicrobial agents.

In conclusion, double-lumen tunnelled CVC is considered appropriate for preparation regimens, medical treatments, stem cell infusions, and parenteral support in autologous and allogeneic stem cell transplantation patients. Our data indicate that the acceptable incidence of complications in our case series did not have a negative effect on neutrophil or thrombocyte engraftment time and did not increase GVHD or mortality rates. However, further prospective multi-centre studies of catheter-related complications and early-period morbidity and mortality are warranted.

## Figures and Tables

**Table 1:**
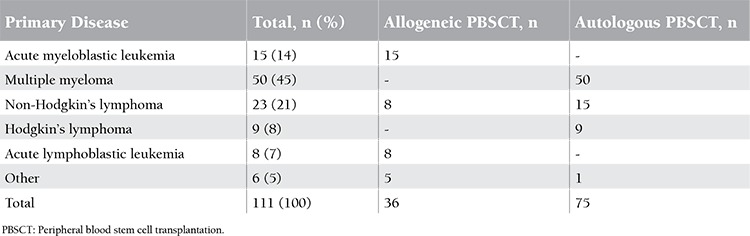
Distribution of patients who underwent catheter placement according to primary disease and haematopoietic stem cell transplantation type.

**Table 2:**
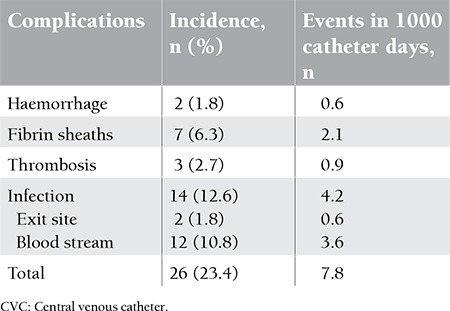
The incidences of tunnelled CVC-related complications in transplant recipients.

**Table 3:**
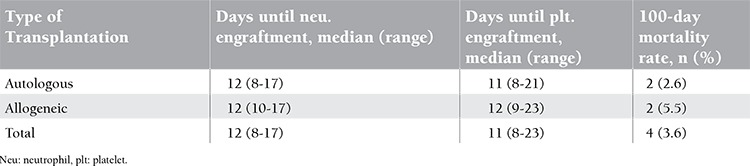
Outcome parameters according to type of stem cell transplantation.
